# Nursing under the M.A.B.

**Published:** 1920-09-18

**Authors:** 


### NURSING UNDER THE M.A.B.

THE M.A.B. is the largest employer of nurses in the kingdom. It has to staff fifteen infectious hospitals, eight institutions for tuberculosis, six mental hospitals, three training colonies for imbeciles and feeble-minded, one colony for sane epileptics, one training-ship infirmary, five children's institutions. Not one of these institutions is capable of giving complete training to nurses; and hence the Board labours under peculiar disadvantages in recruiting the staff required. The recently issued report for 1919-1920 gives a highly interesting summary of the means adopted by the Board to popularise its service.

For the actual recruiting work the Board very sensibly enlisted the assistance of the Ministry of Labour to carry out propaganda work particularly in country districts, and last year they entered into a definite arrangement experimentally with the Ministry to fill vacancies at their various institutions. The plan has proved economical and remarkably efficient, over £1,000 a year previously spent in advertising being saved. During the past year 328 nursing candidates were obtained through the Great Marlborough Street Employment Exchange, where this branch of work is centralised.

We have had reason to comment more than once on the successive rises in salary announced by the Board. It is incumbent on this service to pay higher rates than other institutions for first-year probationers, both on account of the infectious nature of the cases at certain hospitals, and the need for further training before commencing private nursing. These drawbacks are also counterbalanced by the institution of an eight-hour day (or rather a fifty-hour week, with four weeks' holiday), coupled with one day a week off duty. Attention to the comforts of the nurses, an excellent dietary and great improvements in the available accommodation for the staff must also be reckoned among the attractions. The result is highly creditable to the committee which has this branch of organisation in hand, and even more so to the permanent officials.

